# Margarines and Fast-Food French Fries: Low Content of *trans* Fatty Acids

**DOI:** 10.3390/nu9070662

**Published:** 2017-06-28

**Authors:** Iciar Astiasarán, Elena Abella, Giulia Gatta, Diana Ansorena

**Affiliations:** Department of Nutrition, Food Science and Physiology, Faculty of Pharmacy and Nutrition, Universidad de Navarra, C/Irunlarrea s/n, IDISNA—Instituto de Investigación Sanitaria de Navarra, 31008 Pamplona, Spain; iastiasa@unav.es (I.A.); eabella@alumni.unav.es (E.A.); giuliagatta93@gmail.com (G.G.)

**Keywords:** food composition tables, lipid profile, *trans* fat, fast food, spreads

## Abstract

The lipid fraction of margarines and fast food French fries, two types of foods traditionally high in *trans* fatty acids (TFA), is assessed. TFA data reported worldwide during the last 20 years have been gathered and show that some countries still report high TFA amounts in these products. The content of TFA was analysed in margarines (two store and four premium brands) and French-fries from fast-food restaurants (five chains). All samples were collected in Pamplona (Navarra, Spain). The margarines showed mean values of 0.68% and 0.43% (g TFA/100 g fat) for the store and premium brands, respectively. The French fries’ values ranged from 0.49% to 0.89%. All samples were lower than the 2% set by some European countries as the maximum legal content of TFA in fats, and contained less than 0.5 g/serving, so they could also be considered “*trans* free products”. This work confirmed that the presence of TFA is not significant in the two analysed products and contributes updated food composition tables, key tools for epidemiological and nutrition studies.

## 1. Introduction

From 1990 to 2010 globally, the estimated proportional CHD (coronary heart disease) mortality increased by 4% for higher TFA (*trans* fatty acids) consumption, driven basically by increases in low and middle-income countries [1]. As a reaction to the evidences showing a detrimental effect on health caused by the consumption of TFA [2], several countries and organizations established recommendations, limitations, and bans in order to control TFA levels in food [3,4]. It has been stated that removing these fats from the food supply is considered an optimum public health intervention for reducing CVD risk and improving the nutritional quality of diets [5]. Furthermore, these policies have resulted, for instance, in lower concentrations of *trans* fat in Canadian women’s breast milk samples [6] and in lower plasma TFA concentrations in persons with type-2 diabetes [7] and might have contributed to low TFA values in erythrocytes in Europe [8].

The first organisation to suggest a guideline to regulate TFA content in food was the Food and Drug Administration (FDA) in 1999. The proposal established the compulsory inclusion of the amount of TFA on the label of those products in which the content of TFA was higher than 0.5 or more grams (g) per serving. In 2003, Denmark was the first country that introduced a legislation regarding the use of TFA in industrial processed food, limiting the permitted level of TFA to 2 g of TFA per 100 g of fats or oil in the product as sold to the final consumer [9]. This initiative led to a decrease in their CVD mortality rates [10]. On the other hand, in 2009, the World Health Organization (WHO) published a report in which the recommended intake for TFA was set at less than 1% of total energy intake (E) [11]. However, recently this value has been decreased to 0.5% E based on the impact of these fatty acids (FA) on CHD mortality data [1].

The main reason for the food industry to use TFA-containing fats is the interesting characteristics that these type of fatty acids present; solid, edible fat at room temperature, enhanced stability, reduced oxidation susceptibility, and improved organoleptic properties [12].

Since the above-mentioned legislative measures were taken, different strategies to replace TFA have been studied; new processes, treatments, and reformulations such as the modification of the hydrogenation process, interesterification, fractionation, or the combination of several technologies [13,14,15].

In the past few years many efforts have been made to reduce the presence and dietary intake of TFA in foods traditionally rich in TFA such as bakery products, snacks, margarines, fats, and fast foods with mixed success. This is the subject of elegant works carried out in Portugal [16], Pakistan [17], Brazil [18], UK [19], Austria [20], Costa Rica [21], Estonia [22], New Zealand [23], Switzerland [24], Serbia [25], or Lebanon [26]. Our research group conducted a study of bakery products and also in snacks commercialized in Spain and also found very low TFA in these products [27,28]. Regarding fast food, some studies carried out a few years ago reported a high variability among products [29] and even between international fast food companies’ outlets located in different countries [30]. However, other studies carried out during the last few years showed a significant reduction in the amounts of TFA. Thus, monitoring TFA in foods and having updated databases becomes crucial to verify that the progress in the reduction of their presence in diets continues [3,31,32] and that this occurs across all food sectors and countries.

The objective of this work was to test the TFA amounts of different brands of margarines and French fries sold by different well-known fast food chains. These two food items had traditionally high amounts of these fatty acids [33,34]. A contribution to updating food composition tables was also aimed at in this paper. This second objective was in agreement with the proposal of the Spanish Federation of Scientific Societies of Food, Nutrition, and Dietetics (FESNAD), which suggest including TFA content in food labels [33].

## 2. Material and Methods

### 2.1. Materials

Fatty acid methyl esters (individual standards and mixtures of isomers) were purchased from Sigma-Aldrich Chemical^®^ (St. Louis, MO, USA), except for brassidic acid, which was purchased from Nu-Check Prep. Inc. (Elysian, MN, USA). Boron trifluoride/methanol was obtained from Merck^®^ (Whitehouse Station, Hunterdon County, NJ, USA). Methanol, chloroform, petroleum ether, and potassium chloride were from Panreac^®^ (Barcelona, Spain).

### 2.2. Sampling

Margarines: 18 samples from six different brands of well-known and widely distributed margarines were analysed (three of them were three-quarter-fat margarine and three of them half-fat margarine, according to Council Regulation, European Council, 1994) [35]. Among the ¾ Margarines, two of them were store brands (brand 1 and 2) and one of them a premium brand (brand 3). All the half-fat margarines were premium brands. All samples were purchased in regular supermarkets located in Pamplona (Navarra, Spain) and immediately transported to the laboratory, where they were kept in the fridge (4 °C) until analysis. For each brand, three samples (250 g each) from different lots were collected on three different days (*n* = 18).

The following fat sources were declared on the labels of the margarines: Brand 1 had sunflower, rapeseed, and palm oil; Brand 2 had palm and sunflower oil; Brand 3 had sunflower, palm, and linseed oil; Brand 4 had sunflower, olive, palm, and linseed oil; Brand 5 had sunflower, palm, and linseed oil; and Brand 6 had no ingredients reported. The energy values (kcal/100 g) declared for these products were 544, 550, 540, 360, 320, and 316, respectively. Analyses in each batch were performed in triplicate.

French fries: 15 samples of French fries were purchased in the same conditions as customers do in fast food restaurants belonging to well-known international chains (these brands would cover approximately 70% of the Spanish fast food market share. The restaurants were located in Pamplona (Navarra, Spain). After purchase, they were immediately transported to the laboratory, homogenized, and frozen (−20 °C) until further analysis. For each of the five different brands studied (food outlets), three batches (1 kg each) were collected on three different days (*n* = 15). The serving sizes (small portions) reported by the companies for these products were 100 g, 90 g, 103 g, 80 g, and 215 g for brands 1, 2, 3, 4, and 5, respectively. The energy values (kcal/100 g) declared for these products were 323, 258, 223, 290, and 258, respectively. Analyses in each batch were performed in triplicate.

### 2.3. Analysis of Samples

The official method 960.39 of the AOAC [36] was applied for quantitative fat extraction (Soxhlet with Petroleum ether as solvent) (Büchi model B-811 Extraction System, Büchi, Flawil, Switzerland). In order to carry out qualitative fat extraction for the further analysis of the lipid profile, the method described by Folch et al. [37] was followed.

The fatty acid profile was determined in the lipid extracts by gas chromatography [27]. Boron trifluoride/methanol was used for the preparation of fatty acid methyl esters (FAME) [38]. A Perkin-Elmer Autosystem XL gas chromatograph fitted with a capillary column SP^TM^-2560 (100 m × 0.25 mm × 0.2 μm—Sigma Aldrich, St. Louis, MO, USA) and flame ionization detection was used. The temperature of the injection port was 250 °C and of the detector was 260 °C. The oven temperature was programmed at 175 °C for 10 min and increased to 200 °C at a rate of 10 °C/min, then increased to 220 °C at a rate of 4 °C/min, which was kept for 15 min. The carrier gas was hydrogen, and the pressure was 20.5 psi. The split flow was 120 cm/s. The identification of the fatty acid methyl esters was done by the comparison of the retention times of the peaks in the sample with those of standard pure compounds. Individual methylated standards were used for all fatty acids except for the following mixtures of isomers: linoleic acid isomers (mixture 47791), linolenic acid isomers (mixture 47792), and mixtures 20:1, 20:2, 20:4, and 20:5 (mixture 18912), all from Sigma-Aldrich (St. Louis, MO, USA). The order of elution in the case of the mixtures of isomers (linoleic and linolenic acid *cis*/*trans* isomers) was also taken into account (Sigmaldrich.com—FAME Application guide) [39,40]. Spiking the sample with each individual standard (or mixture of standards in the case of the linoleic or linolenic acid isomers and the C20 mixture) was finally used for confirming the identification. The quantification of individual fatty acids was based on the internal standard method using heptadecanoic acid methyl ester (Sigma-Aldrich, St. Louis, MO, USA). Elaidic acid (9t-18:1) eluted very closely to other 18:1 *trans* isomers (possibly t6 to t12), which were all located earlier than oleic acid (9c-18:1). Quantification for all these 18:1 *trans* isomers was done as the sum of all of them. The chromatograms obtained for margarine and French fries are shown as supplementary material (Figures S1 and S2, respectively).

## 3. Statistical Analysis

Statistical analyses were performed with Stata IC 12 software (Copyright 1985–2011 StataCorp LPm, Revision 2014, College Station, TX, USA). The mean and standard deviation of data are reported in the tables. The median, 25th, and 75th percentiles are shown in the figures. A one-way ANOVA test (Analysis of variance) followed by a post-estimation test (Bonferroni) were used to determine significant differences among the different brands. A significance level of *p* ≤ 0.05 was used for all evaluations. The Pearson correlation test was applied for the evaluation of the association between polyunsaturated fatty acids (PUFA) and TFA content in French fries.

## 4. Results and Discussion

Information on *trans*-fat intake in several countries of the WHO European Region is still very limited, and a large number of products containing high levels of *trans* fat are still available on the market [5]. A display of the TFA content of foods in the Nutrition Facts table is not mandatory according to current legislation in Europe, and therefore consumers and health-related professionals are not provided with information on the levels of TFA in products. Thus, this work reports updated lipid composition data on two products included within the list of foodstuffs susceptible to containing TFA [34]. For both types of products (margarines and French fries), the lipid profile was expressed in g/100 g fat and also in g/100 g product in order to discuss the differences found with previous studies and also to estimate the intake of TFA in diets.

Table 1 gathers the lipid composition data of margarines and French fries reported by different research groups during the last decades. Regarding margarines, although a general trend towards an average reduction in the TFA content was observed, large differences between countries were noticed. Also, Stender et al. [41] pointed out wide differences in the TFA content of several popular foods in Europe. These are probably linked to the different types of interventions to reduce the intake of industrially produced TFA worldwide (legislative limits or voluntary policy programs), showing certain variability in their success [42]. In 2003, the first regulation on TFA content in foods came into force in Denmark. Since then, only seven out of 37 types of margarines and spreads analysed all over the world and presented in Table 1 showed mean values lower than 2% TFA. Moreover, in the last five years, values of up to 30% TFA of total fat were still observed in some European margarine samples [22,25]. In Spain, the last data published on these products showed an average TFA content of 2.8 g/100 g fat [43]. Since then, a careful reformulation of products was undertaken by the Spanish industry, evident by the data provided in this study.

Far fewer studies reported the TFA content in French fries (Table 1). Although the results showed great diversity, there was a trend towards a lower TFA content is these products in the last few years.

### 4.1. Analysed Margarines

**Figure 1:**
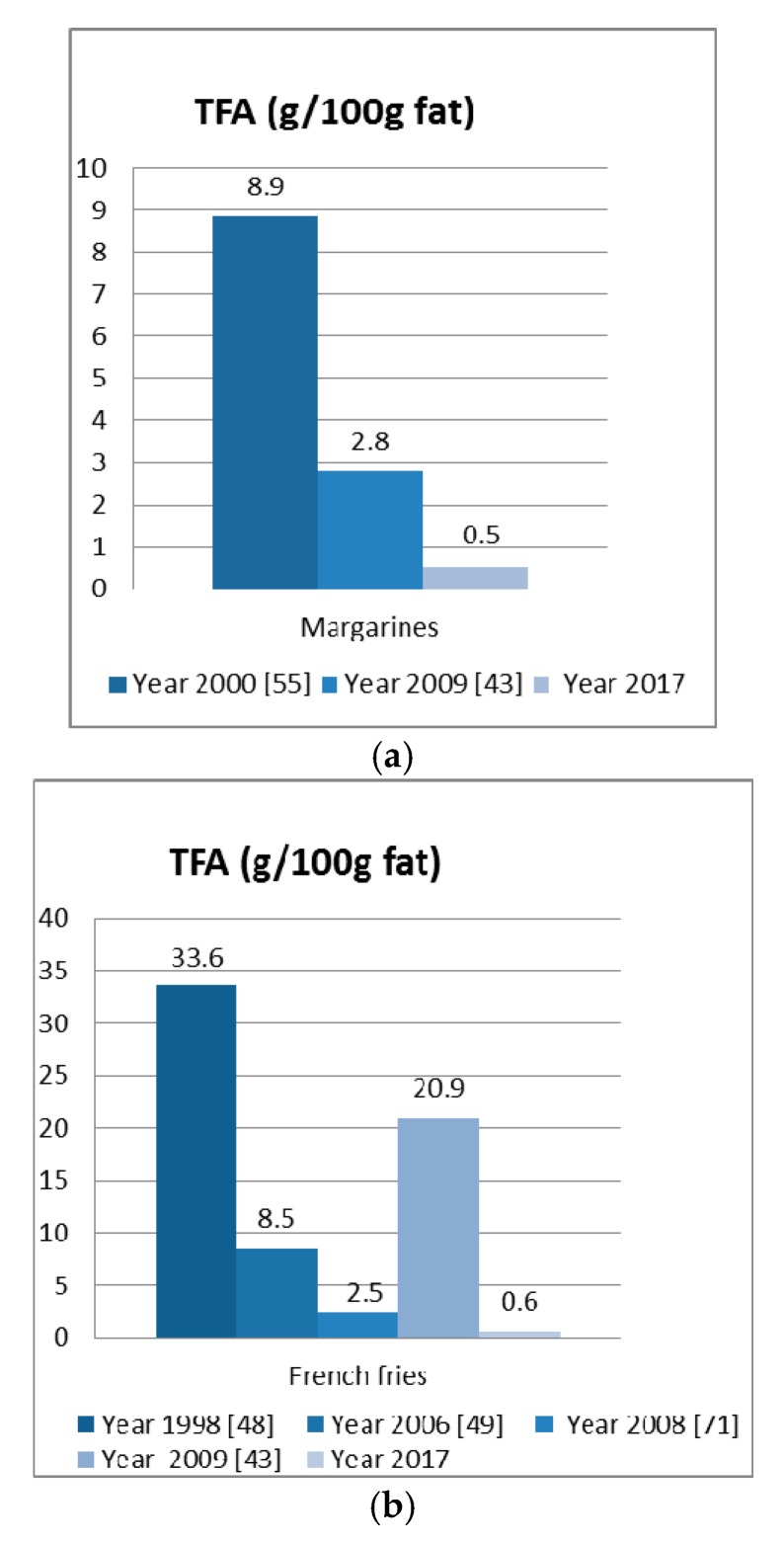
Evolution of the TFA content (g/100 g fat) in (**a**) margarines and (**b**) French fries in Spain. Between brackets, the reference of the paper is indicated.

The detailed lipid profile (g/100 g fatty acids) of the six types of margarines analysed in this study is shown in Table 2. The mean value for the six brands was significantly lower (0.51 g/100 g) than data reported by previous studies in Spain. Figure 1a shows the differences between this value and those obtained from previous studies (in the year 2000 [44] and the year 2009 [43]). The minimum value found in our work was 0.29 g/100 g (the mean for brand 4), and the maximum one was 0.72 g/100 g (the mean for brand 2). A comparison of the ¾ to the ½ fat containing margarine showed, in general, that the former had a higher proportion of TFA. However, brand 3 (premium brand), showed similar TFA values to the ½ fat margarines. Based on these results, it was concluded that there was, on average, a 5.5-fold reduction in TFA content compared to the data published in 2009 [43].

Remarkably, none of the samples reached the 2% value set by those European countries in which a *trans*-fat policy is currently in place by legislation (Denmark, Switzerland, Austria, Iceland, Hungary, and Norway). None of the samples declared partially hydrogenated vegetable oils among their ingredients, which is in agreement with our results. The current results were consistent with those found for two margarines analysed by ATR-FTIR (attenuated total reflection-Fourier transform infrared) spectroscopy in Spain [45].

In all cases, the solution to reach a compromise between a healthier lipid profile and the desired textural properties for these products was adequately addressed without the use of partially hydrogenated oils. A complete lipid analysis of the margarine showed that oleic acid was the most abundant fatty acid in three out of six brands, which suggested that olive oil or rapeseed oil was used in their formulations. In fact, brand 1 (store brand) showed the highest MUFA and lowest *n*-6/*n*-3 ratio among all the tested margarines, which would suggest that rapeseed was included in this product. In brands 2, 3, and 5, linoleic acid was the major fatty acid, which is consistent with the use of sunflower oil in their formulae. Relevant percentages of alpha-linolenic acid were also noticed (up to 8.68% in brand 5) as a consequence of the incorporation of linseed oil in the fat blends. When this oil was present in the blends (in brands 3, 4, and 5), it significantly contributed to obtaining *n*-6/*n*-3 ratios of 4.7 to 5.9. However, it did not contribute *trans* linolenic isomers.

The use of palm oil is one of the technological strategies currently applied to reduce the TFA content in margarines without drastic modification of their textural properties. Its saturated fatty acids (SFA) content and high melting point make this oil a good alternative for that purpose. Palm oil was present in all the analysed products, which resulted in increased levels of palmitic acid from 13% (brand 3) to 19% (brand 1). The incorporation of palm oil into the final blends was well balanced from the nutritional perspective. The total SFA content of the new margarines is lower (Table 2) than in the high TFA margarines of the past, wherein the mean value of SFA plus TFA was generally 40 g/100 g fat (Table 1), a value that even exceeded the content of MUFA or PUFA in the earlier products. Furthermore, according to current European nutrition claims regulation (Regulation 1924/2006) [70], brands 3 and 5 could claim “high in polyunsaturated fat” (with >45% PUFA content and PUFA providing >20% total energy of the product).

Table 3 shows the lipid profile data expressed over g/100 g product. Brands 1 to 3 contained approximately 60 g fat/100 g and led to higher TFA values (up to 0.44 g/100 g product) than brands 4 to 6, which contained only 40% fat and lower TFA values (up to 0.22 g/100 g). When comparing these data to the limit set by the USA regulation to consider a “*trans* free product” (0.5 g TFA/serving), all of them complied with this condition, including the store brands, with values from 0.02 to 0.07 g/serving (serving size = 15 g). SFA content, which should be declared in the nutrition facts table, ranged from 9.66 g/100 g product (brand 5) to 16.72 g/100 g (brand 2). Thus, taking into account these data, one serving of the analysed products would supply between 0.65 and 1.12% of an individual’s total energy value, which can be considered low, taking into account the suggestion to limit the intake of SFA to less that 10% of total energy [1].

### 4.2. Analysed French Fries

The frying process implies degradation of oils and fats, leading to numerous fatty acid alteration products that are absorbed by the fried foods. Among the degradation products are *trans* fatty acids formed as a consequence of the geometrical isomerization of double bonds [71]. In this sense, it has been described that the repeated use of frying oil for two weeks resulted in a significant increase in TFA content in the extracted oils from French fries purchased in fast food restaurants [69]. It was for this reason that partially hydrogenated oils were generally used for deep fat frying because of the stability of these lipids against oxidative deterioration during frying.

For some time now, frying methods and oils used have been progressively altered to minimize TFA presence in French fries. As is evident in Table 1, better frying practices led to decreased TFA content from values around 30% in 1998 [64], and even up to 42% in 2006 [65], to values lower than 2% in more recent studies [17,19,69].

Our data confirmed the reduction of TFA in French fries available from five Spanish fast food chains (Table 4). The mean value for the five brands was 0.61 g/100 g, which is significantly lower than the data reported by previous studies in Spain. Figure 1b shows the differences between this data and that obtained from earlier studies (1998 [64] and 2006 [65], 2008 [66] and 2009 [43]).

Brand 4, the highest in TFA (0.89 g/100 g), was characterized by a higher linoleic and lower oleic content compared to the rest of brands. Oleic was by far the most abundant fatty acid in brands 1, 2, 3, and 5 (mean value 64.13%), while linoleic acid was the most abundant acid in brand 4 (51.03%). These data were related to another remarkable difference of brand 4, which was the relative abundance of the *trans* 18:2 to the *trans* 18:1 FA isomer; only 30% of the total TFA derived from C18:1 isomers, with C18:2 isomers being the major contributors to this type of lipid (64%). The high oleic acid content in four of the five brands analysed could be explained by the use of high oleic sunflower oil (probably in brands 1 and 5, in which very low values of palmitic acid were noticed) or by the use of olive oil or blends of high oleic sunflower with palm oil (brands 2 and 3, with palmitic around 14%).

Also, a noticeable reduction in the total fat content of the French fries was observed in this study (Table 5) compared to previous reports. Fernández-San Juan [43] reported an average fat content of 20.4 g/100 g, whereas, in our study, the percentage of fat for different brands ranged from 8.32% (brand 3) to 14.58% (brand 1). This decrease in the fat content led to the significantly lower energy value of these products, which was a positive consequence, as high energy content has been one of the drawbacks that has traditionally been pointed out for fast food French fries [72].

Expressing the TFA content in g/100 g product (Table 5) led to very low values, ranging from 0.04 to 0.11 g TFA/100 g product. It has to be mentioned that a significant correlation was found between the PUFA content and the TFA content (Pearson *R* = 0.6; *p* < 0.001), whereas no association was found for TFA and MUFA, pointing to a higher susceptibility of *trans* fat formation from PUFA than from MUFA. Using the serving size criteria, very low values were also found (lower than 0.11 g TFA/serving), which allowed all these products to claim “*trans* free product”. On the other hand, the energy supply from the high MUFA content in brands 1, 2, 3, and 4 (>20% of total energy) allowed these products to claim ‘high monounsaturated fat’ according to the current European Regulation 1924/2006.

**Figure 2:**
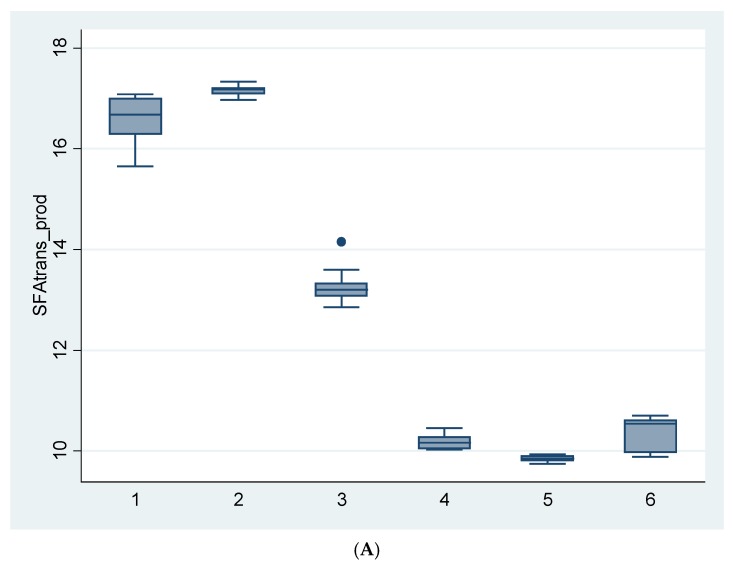

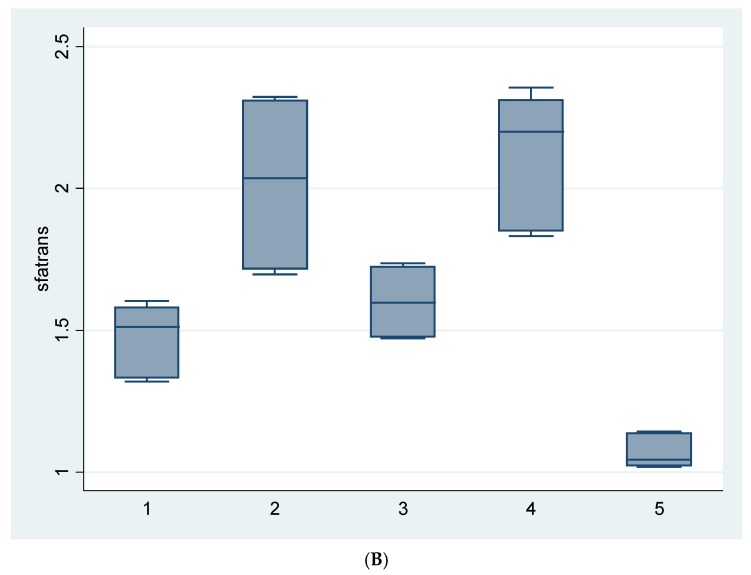
Boxplot for the sum of SFA plus *trans* fat (g/100 g product) for all samples analyzed. The median, 25th, and 75th quartiles and the maximum and minimum values are represented. SFA: sum of saturated fatty acids. (**A**) Margarines and (**B**) French fries.

Considering the well-known implications of SFA and TFA on coronary heart disease, the optimal dietary intake of SFA is set at <10% of the total energy value (E), whereas the optimal intake for TFA is set at <0.5% E [1]. The sum of both types of fatty acids (g/100 g product) is presented in Figure 2. In the case of margarines, the premium brands showed lower total values compared to the store brands. In these samples, the sum of SFA plus TFA (9.8–17.2 g/100 g product) supplied between 22–29.5% of the total energy of the food. However, in the case of French fries, the contribution of SFA plus TFA (1.1–2.1 g/100 g product) to the total energy value was very low (4–7%). These data pointed out that the decrease in TFA content did not lead to a significant increase in SFA content and that the sum of both fractions resulted in much lower values than those found in the past. Nevertheless, the daily average intake of these two products should be taken into account to determine their contribution of these amounts to the total diet.

### 4.3. Conclusions

Food frequency questionnaires and food composition tables are two remarkable and useful tools in many epidemiological and interventional studies, the role of which is to provide accurate information and detect appropriate targets for public health nutrition campaigns. According to Brownell and Pomeranz [73], the government has the authority and responsibility to regulate the unhealthful aspects of the food supply, and this should be done while relying on accurate data. This paper reported data on Spanish margarines (premium and store brands) and fast-food French fries from five well-known international chains, showing the very low TFA contents of both types of products. In the case of margarines, the low TFA content was achieved using appropriate oil blends, and, in the case of French fries, the reduction was due to the combined effect of using better quality oils and applying frying methods that result in a lower total fat content in the product. Interestingly, the decrease in TFA was not linked to increments in the SFA content.

## Figures and Tables

**Table 1:** Evolution of the *trans* fat content in margarines and French fries worldwide (references in chronological order).

Authors and Country of the Work	Ref.	Notes	%Fat	g TFA/100 g Fat	g TFA/100 g Product	g TFA + SFA/100 g Fat
**Margarines**						
Lake et al. (1996)–New Zealand	[46]	Margarines	68.9	**16.4 (12.6**–**19.7)**	11.3 (8.7–13.6)	
		Table spreads	37.38	**15.7 (14.3**–**16.9)**	5.9 (5.3–6.3)	
		Butter/margarine blends	64.42	9.6 (6.1–**13.1**)	6.2 (3.9–8.4)	
Ratnayake et al. (1998)–Canada	[47]	Tub margarines from non hydrogenated VO	-	2.2 (0.9–5.0)	-	-
		Tub margarines from partially hydrogenated VO	-	**21.4 (10.5**–**44.8)**	-	-
		Print margarines from partially hydrogenated VO	-	**34.3 (16.3**–**47.7)**	-	-
Wagner et al. (2000)–Austria	[48]	Margarines	-	1.6 (0.3–3.73)	-	-
Alonso et al. (2000)–Spain	[44]	Margarines	63.5	8.87 (0.40–**21.28**)	5.63	35.03 (27.4–45.27)
Tavella et al. (2000)–Argentina	[49]	Margarines	82	**25.38**	20.81	-
		Margarines	50	**31.84**	15.92	-
Tekin et al. (2002)–Turkey	[50]	Margarines - Stick	-	**19** (0–**37.8**)	-	56.3
Marekov et al. (2002)–Bulgaria	[51]	Margarines	80	1.6	1.28	28.5
Karabulut & Turan (2006)–Turkey	[52]	Shortenings	-	8.95 (2–**16.5**)		50.8
Baylin et al. (2007)–Costa Rica	[21]	Stick regular	-	**13.25**	-	50.47
Ratnayake et al. (2007)–Canada	[53]	Tub margarines from non hydrogenated VO	-	0.8 (0.5–1.7)	-	-
		Tub margarines from partially hydrogenated VO	-	**20 (17.0**–**32.6)**	-	-
		Print margarines from partially hydrogenated VO	-	**39.3 (39.2**–**42.9)**	-	-
McCarthy et al. (2008)–Australia	[54]	Hard margarines	90.4 (80.5–100)	3.2 (1.7–4.5)	3.0 (1.7–4.5)	-
		Soft margarines	64.6	**11.6**	7.5	-
Haytowitz et al. (2008)–USA	[55]	Margarines -Data 2002	80	**19.69**	13.3	-
		Margarines -Data 2006	80	**14.7 (11.2**–**18.8)**	11.8 (9–15.04)	-
Wagner et al. (2008)–Austria	[20]	Household margarines	80	1.45 ± 1.99	1.16 ± 1.57	-
		Industrial margarines	79.3	7.83 ± 9.97	6.21 ± 7.84	-
Fu et al. (2008)–China	[56]	Margarines	77.29 (25.02–99.81)	5.09 (0–**13.21**)	3.9 (0–10.21)	-
Saunders et al. (2008)–New Zealand	[23]	Margarines and table spreads	64.9 (58.9–72.1)	5.3 (2.7–6.9)	3.4 (1.7–4.5)	32.8
Basol & Tasan (2008)–Turkey	[57]	Shortenings	-	**12.6** (2.7–**23.9**)	-	52.3
		Margarines	-	**11.24** (0–**39.4**)	-	44.94
Albers et al. (2008)–USA	[58]	Margarines	56.4	6.6 ± 10.4	3.7	25.5 ± 11.1
Fernández-San Juan (2009)–Spain	[43]	Margarines		2.8 ± 1.7		
Richter et al. (2009)–Switzerland	[24]	Semi-solid fats	-	3.86 (0.1–**29.3**)	-	-
Cavendish et al. (2010)–Brazil	[59]	Hydrogenated margarines	50	**15.8**	7.91 ± 1.05	39.42
		Hydrogenated margarines	20	**12.3**	2.46 ± 0.39	34.8
		Interesterified margarines	65	1.98	1.29 ± 0.47	27.46
		Interesterified margarines	30	2.17	0.65 ± 0.24	26.07
Kuhnt et al. (2011)–Germany	[60]	Houlsehold margarines	70.6	0.96 (0.11–4.28)	0.7 (0.08–3.0)	33.99
		Industrial margarines	87.8	1.70 (0.05–**14.38**)	1.5 (0.04–12.6)	47.06
Pajin et al. (2011)–Serbia	[61]	Puff pastry margarines	-	7.7 (0.68–**23.77**)	-	53.73
Hernández-Martínez et al. (2011)–Mexico	[62]	Spreadable margarines	95	5.35 (0–**20.66**)	5.12 (0–19.68)	29.35
		Stick margarines		8.71 (0.25–**23.87**)	8.34 (0.24–22.84)	49.63
Meremae et al. (2012)–Estonia	[22]	Margarines	47.6	3.84 (0.04–**34.96**)	1.83	31.38
		Shortenings	70.62	6.11 (0.14–**39.50**)	4.31	48.39
		Blended spreads	65.7	2.97 (1.18–9.08)	1.95	61.64
Roe et al. (2013)–UK	[19]	Margarine hard block	76.4	0.1	0.08	-
		Fat spread (41–62% fat)	59.6 (59.1–60.6)	0.22 (0.19–0.25)	0.13 (0.11–0.15)	-
		Fat spread (26–39% fat)	38.3 (36.9–39.0)	0.28 (0.15–0.38)	0.10 (0.06–0.14)	-
Vucic et al. (2016)–Serbia	[25]	Hard margarines	80	4.5–**28.8**	3.6–23.4	58.8 (57.03–63.84)
		Soft margarines	50 (25-60)	2.28 (0.17–6.89)	0.08–3.4	31.5 (23.33–42.36)
Costa et al. (2016)–Portugal	[63]	Margarines and shortenings	-	0.83 (0.26–2.16)	0.56 (0.16–1.57)	-
**French Fries**						
Aro et al. (1998)–various countries	[64]	French fries	14.3 (11–18)	**12**–**35 33.57** in Spain	1.7	-
Wagner et al. (2000)–Austria	[48]	French fries		1.9–18	3	
Stender et al. (2006)–various countries	[65]	French fries	-	1 (Germany, Denmark) 4–**13** (Spain) **28** (South Africa) **42** (Poland)	-	-
Barrado et al. (2008)–Spain	[66]	French fries	35.84 (over dry matter)	2.5	-	31.4
McCarthy et al. (2008)–Australia	[54]	French fries	17.3 (11.7–21.4)	2.1 (1.4–3.4)	0.3 (0.3–0.4)	-
Wagner et al. (2008)–Austria	[20]	French fries		1.72 ± 3.04	0.30 ± 0.55 (up to 1.6)	
Fernández-San Juan (2009)–Spain	[43]	French fries	20.4	**20.9** ± 12.9	4.3	13.1
Fritsche et al. (2010)–Germany	[67]	French fries	22.5	0.2 (0.09–0.3)	0.04	
Tyburczy et al. (2012)–USA	[40]	French fries	14.7 (12.14–17.50)	6.6 (0.6–**12.62**)	0.97	27.53
Chung et al. (2013)–Hong Kong	[68]	Potato products (French fries, wedges, chips)	26 (12–39)	0.5 (0.13–1.46)	0.13 (0.035–0.38)	6.93
Roe et al. (2013)–UK	[19]	Potato chips, takeaway (fish and chips shop)	8.4	2.05	0.17	-
		Potato chips, fine cut, takeaway (fast food outlets)	14.2	0.11	0.01	-
Karim et al. (2014)–Pakistan	[17]	French fries	-	0.11–**24**	-	-
Yildirim et al. (2015)–Turkey	[69]	French fries	-	1.24 (0–7.19)	-	41.43 (24.41–49.33)

In bold, values higher than 10 g *trans* fatty acids (TFA)/100 g fat are highlighted.

**Table 2:** The fatty acid profile (g/100 g fat) for the ¾ fat margarines (brands 1, 2, and 3) and for the half-fat margarines (brands 4, 5, and 6) are analyzed (mean and standard deviation).

Fatty Acid	Brand 1		Brand 2		Brand 3		Brand 4		Brand 5		Brand 6	
	Mean	SD	Mean	SD	Mean	SD	Mean	SD	Mean	SD	Mean	SD
Caprylic 8:0	0.30	0.02 ^a^	0.47	0.0 ^c^	0.30	0.02 ^a^	0.31	0.03 ^a^	0.39	0.02 ^b^	0.32	0.02 ^a^
Capric 10:0	0.30	0.03 ^a^	0.41	0.04 ^c^	0.29	0.02 ^a^	0.30	0.03 ^a^	0.37	0.02 ^b^	0.31	0.01 ^a^
Lauric 12:0	2.76	0.06 ^c^	2.58	0.13 ^b^	2.70	0.09 ^b,c^	2.91	0.21 ^d^	2.73	0.03 ^c^	2.28	0.03 ^a^
Myristic 14:0	1.28	0.06 ^d^	1.37	0.06 ^e^	1.13	0.06 ^b^	1.20	0.03 ^c^	1.16	0.01 ^b,c^	1.07	0.01 ^a^
Palmitic 16:0	18.80	0.44 ^f^	18.52	0.13 ^e^	12.88	0.31 ^a^	15.71	0.10 ^c^	14.31	0.12 ^b^	18.04	0.23 ^d^
*t*-Palmitoleic 9*t*-16:1	0.02	0.02 ^b^	0.01	0.01 ^a^	0.01	0.01 ^a^	0.00	0.00 ^a^	0.00	0.00 ^a^	0.00	0.00 ^a^
Palmitoleic 9*c*-16:1	0.18	0.01 ^b^	0.12	0.01 ^a^	0.10	0.00 ^a^	0.25	0.05 ^c^	0.12	0.00 ^a^	0.28	0.01 ^d^
Stearic 18:0	2.62	0.10 ^a^	3.63	0.16 ^c^	3.82	0.07 ^d^	3.71	0.05 ^c^	3.64	0.03 ^c^	3.38	0.04 ^b^
Σ *trans* 18:1	0.37	0.06 ^e^	0.22	0.19 ^d^	0.10	0.01 ^b,c^	0.05	0.06 ^a^	0.06	0.01 ^a,b^	0.11	0.01 ^c^
Oleic 9*c*-18:1	45.40	0.99 ^f^	29.60	0.79 ^c^	25.11	1.18 ^a^	35.28	0.81 ^d^	26.11	0.20 ^b^	39.84	0.86 ^e^
11*c*-18:1	2.26	0.18 ^c^	0.65	0.09 ^a^	0.70	0.11 ^a,b^	0.83	0.05 ^b^	0.60	0.04 ^a^	0.59	0.23 ^a^
*t*-Linoleic 9*t*, 12*t*-18:2	0.00	0.01 ^a^	0.00	0.01 ^b^	0.01	0.01 ^b^	0.00	0.00 ^a^	0.02	0.00 ^b^	0.01	0.00 ^b^
*c-t* Linoleic 9*c*, 12*t*-18:2	0.12	0.01 ^a^	0.26	0.00 ^e^	0.16	0.00 ^b^	0.13	0.00 ^a^	0.19	0.01 ^c^	0.23	0.01 ^d^
*t-c* Linoleic 9*t*, 12*c*-18:2	0.12	0.01 ^a,b^	0.23	0.01 ^d^	0.13	0.01 ^b^	0.12	0.01 ^a^	0.18	0.01 ^c^	0.22	0.01 ^d^
Linoleic 9*c*, 12*c*-18:2	17.30	1.74 ^a^	40.92	0.94 ^d^	46.11	1.12 ^d^	33.82	0.73 ^c^	40.69	0.11 ^d^	32.13	1.01 ^b^
Arachidic 20:0	0.43	0.05 ^d^	0.17	0.06 ^b,c^	0.16	0.04 ^b^	0.21	0.03 ^c^	0.07	0.03 ^a^	0.20	0.02 ^b,c^
γ-Linolenic 18:3*n*-6	0.38	0.07 ^c^	0.00	0.00 ^a^	0.17	0.04 ^b^	0.00	0.00 ^a^	0.20	0.06 ^b^	0.02	0.02 ^a^
Eicosenoic 20:1	0.73	0.21 ^b^	0.13	0.01 ^a^	0.09	0.01 ^a^	0.12	0.02 ^a^	0.08	0.01 ^a^	0.15	0.02 ^a^
α-Linolenic 18:3*n*-3	5.95	0.40 ^d^	0.19	0.06 ^a^	5.88	0.20 ^d^	4.76	0.14 ^c^	8.68	0.12 ^e^	0.59	0.09 ^b^
Eicosadienoic 20:2	ND		ND		ND		ND		ND		ND	
Behenic 22:0	0.25	0.04 ^b^	0.44	0.23 ^c^	0.50	0.03 ^d^	0.41	0.10 ^c^	0.45	0.11 ^c^	0.02	0.0 ^a^
Brassidic 13*t*-22:1	ND		ND		ND		ND		ND		ND	
Erucic 22:1	ND		ND		ND		ND		ND		ND	
Eicosatrienoic 20:3*n*-3	ND		ND		ND		ND		ND		ND	
Arachidonic 20:4*n*-6	ND		ND		ND		ND		ND		ND	
Eicosapentaenoic 20:5*n*-3	ND		ND		ND		ND		ND		ND	
Nervonic 24:1	0.09	0.01 ^b^	0.00	0.00 ^a^	0.00	0.00 ^a^	0.00	0.00 ^a^	0.00	0.00 ^a^	0.00	0.00 ^a^
Docosatrienoic 22:3	0.07	0.00 ^b^	0.00	0.00 ^a^	0.00	0.00 ^a^	0.00	0.00 ^a^	0.00	0.00 ^a^	0.00	0.00 ^a^
Docosapentaenoic 22:5*n*-6	ND		ND		ND		ND		ND		ND	
Lignoceric 24:0	ND		ND		ND		ND		ND		ND	
Docosapentaenoic 22:5*n*-3	0.00	0.00 ^a^	0.04	0.00 ^a,b^	0.00	0.00 ^a^	0.05	0.00 ^a^	0.06	0.00 ^a^	0.15	0.02 ^c^
Docosahexaenoic 22:6*n*-3	ND		ND		ND		ND		ND		ND	
SFA	26.73	0.64 ^e^	27.60	0.16 ^f^	21.79	0.47 ^a^	24.69	0.36 ^c^	23.06	0.11 ^b^	25.62	0.23 ^d^
MUFA	48.86	1.27 ^e^	30.52	0.86 ^b^	26.00	1.28 ^a^	36.38	0.82 ^c^	26.85	0.16 ^a^	40.90	1.05 ^d^
PUFA	23.77	1.42 ^a^	41.16	0.85 ^d^	52.17	1.32 ^f^	38.64	0.70 ^c^	49.63	0.14 ^e^	32.92	1.07 ^b^
*n*-3	5.95	0.39 ^d^	0.23	0.11 ^a^	5.88	0.20 ^d^	4.82	0.18 ^c^	8.74	0.11 ^e^	0.76	0.10 ^b^
*n*-6	17.69	1.67 ^a^	40.93	0.95 ^d^	46.28	1.13 ^e^	33.83	0.71 ^c^	40.90	0.12 ^d^	32.16	1.02 ^b^
*n*-6/*n*-3	3.00	0.49 ^a^	226.96	119.21 ^b^	7.87	0.10 ^a^	7.04	0.31 ^a^	4.69	0.06 ^a^	42.71	4.52 ^a^
PUFA + MUFA/SFA	2.72	0.09 ^b^	2.60	0.02 ^a^	3.59	0.07 ^f^	3.04	0.08 ^d^	3.32	0.02 ^e^	2.88	0.03 ^c^
TFA	0.64	0.03 ^e^	0.72	0.02 ^f^	0.41	0.04 ^b^	0.29	0.04 ^a^	0.45	0.01 ^c^	0.57	0.02 ^d^

Different letters in the same row indicate significant differences (*p* < 0.05) among the mean values for the different brands. ND: Not detected; SFA: sum of all saturated fatty acids; MUFA: sum of all monounsaturated fatty acids; TFA: sum of all *trans* fatty acids.

**Table 3:** Fatty acid profile (g/100 g product) and total fat content (g/100 g product) for the ¾ fat margarines (brands 1, 2, and 3) and for the half-fat margarines (brands 4, 5, and 6) analyzed (mean and standard deviation).

Fatty Acid	Brand 1		Brand 2		Brand 3		Brand 4		Brand 5		Brand 6	
	Mean	SD	Mean	SD	Mean	SD	Mean	SD	Mean	SD	Mean	SD
Caprylic 8:0	0.18	0.01 ^b^	0.29	0.03 ^c^	0.18	0.01 ^b^	0.12	0.01 ^a^	0.16	0.01 ^b^	0.13	0.01 ^a^
Capric 10:0	0.18	0.01 ^c^	0.25	0.02 ^d^	0.17	0.01 ^b^	0.12	0.01 ^a^	0.16	0.01 ^b^	0.12	0.00 ^a^
Lauric 12:0	1.67	0.04 ^c^	1.56	0.08 ^c^	1.61	0.06 ^c^	1.18	0.09 ^b^	1.14	0.01 ^b^	0.90	0.03 ^a^
Myristic 14:0	0.77	0.03 ^d^	0.83	0.04 ^e^	0.67	0.02 ^c^	0.49	0.02 ^b^	0.48	0.02 ^b^	0.42	0.01 ^a^
Palmitic 16:0	11.39	0.37 ^e^	11.23	0.07 ^e^	7.69	0.22 ^d^	6.40	0.23 ^b^	8.98	0.05 ^a^	7.15	0.25 ^c^
*t*-Palmitoleic 9*t*-16:1	0.01	0.01 ^b^	0.01	0.00 ^b^	0.01	0.00 ^b^	0.00	0.00 ^a^	0.00	0.00 ^a^	0.00	0.00 ^a^
Palmitoleic 9*c*-16:1	0.11	0.02 ^d^	0.07	0.00 ^b^	0.05	0.01 ^a^	0.10	0.02 ^c^	0.05	0.00 ^a^	0.10	0.01 ^c^
Stearic 18:0	1.58	0.05 ^b^	2.20	0.09 ^d^	2.28	0.05 ^c^	1.51	0.02 ^b^	1.52	0.01 ^b^	1.34	0.05 ^a^
Σ *trans* 18:1	0.22	0.04 ^e^	0.13	0.01 ^d^	0.06	0.01 ^c^	0.02	0.02 ^a^	0.03	0.00 ^a,b^	0.04	0.00 ^b,c^
Oleic 9*c*-18:1	27.52	0.96 ^e^	17.93	0.46 ^d^	14.99	0.81 ^b^	14.33	0.33 ^b^	10.92	0.08 ^a^	15.78	0.61 ^c^
11*c*-18:1	1.37	0.12 ^c^	0.39	0.05 ^b^	0.42	0.07 ^b^	0.34	0.02 ^b^	0.25	0.01 ^a^	0.23	0.09 ^a^
*t*-Linoleic 9*t*, 12*t*-18:2	0.00	0.00 ^a^	0.00	0.00 ^a^	0.01	0.01 ^b^	0.00	0.00 ^a^	0.01	0.00 ^b^	0.01	0.00 ^a,b^
*c-t* Linoleic 9*c*, 12*t*-18:2	0.07	0.00 ^c^	0.16	0.00 ^e^	0.09	0.00 ^d^	0.05	0.00 ^a^	0.08	0.00 ^b^	0.09	0.00 ^d^
*t-c* Linoleic 9*t*, 12*c*-18:2	0.08	0.01 ^c^	0.14	0.00 ^d^	0.08	0.01 ^b^	0.05	0.01 ^a^	0.08	0.00 ^b^	0.09	0.00 ^c^
Linoleic 9*c*, 12*c*-18:2	10.47	0.90 ^a^	24.80	0.60 ^e^	27.53	0.83 ^f^	13.78	0.29 ^c^	17.05	0.04 ^d^	12.70	0.42 ^b^
Arachidic 20:0	0.26	0.30 ^c^	0.10	0.03 ^b^	0.10	0.03 ^b^	0.09	0.01 ^b^	0.03	0.01 ^a^	0.08	0.01 ^b^
γ-Linolenic 18:3*n*-6	0.23	0.05 ^c^	0.00	0.00 ^a^	0.10	0.02 ^b^	0.00	0.00 ^a^	0.09	0.02 ^b^	0.01	0.01 ^a^
Eicosenoic 20:1	0.48	0.04 ^d^	0.08	0.00 ^c^	0.05	0.01 ^b^	0.05	0.01 ^a,b^	0.03	0.00 ^a^	0.06	0.01 ^b,c^
α-Linolenic 18:3*n*-3	3.61	0.29 ^c^	0.11	0.04 ^a^	3.51	0.15 ^c^	1.93	0.06 ^b^	3.63	0.05 ^c^	0.23	0.03 ^a^
Eicosadienoic 20:2	ND	ND	ND	ND	ND	ND						
Behenic 22:0	0.15	0.02 ^b^	0.27	0.03 ^d^	0.30	0.02 ^e^	0.17	0.01 ^b,c^	0.19	0.00 ^c^	0.01	0.03 ^a^
Brassidic 13*t*-22:1	ND		ND		ND		ND		ND		ND	
Erucic 22:1	ND		ND		ND		ND		ND		ND	
Eicosatrienoic 20:3*n*-3	ND		ND		ND		ND		ND		ND	
Arachidonic 20:4*n*-6	ND		ND		ND		ND		ND		ND	
Eicosapentaenoic 20:5*n*-3	ND		ND		ND		ND		ND		ND	
Nervonic 24:1	0.05	0.04 ^b^	0.00	0.00 ^a^	0.00	0.00 ^a^	0.00	0.00 ^a^	0.00	0.00 ^a^	0.00	0.00 ^a^
Docosatrienoic 22:3	0.05	0.01 ^b^	0.00	0.00 ^a^	0.00	0.00 ^a^	0.00	0.00 ^a^	0.00	0.00 ^a^	0.00	0.00 ^a^
Docosapentaenoic 22:5*n*-6	ND		ND		ND		ND		ND		ND	
Lignoceric 24:0	ND		ND		ND		ND		ND		ND	
Docosapentaenoic 22:5*n*-3	0.00	0.00 ^a^	0.02	0.00 ^b^	0.00	0.00 ^a^	0.02	0.00 ^b^	0.02	0.00 ^b^	0.06	0.00 ^c^
Docosahexaenoic 22:6*n*-3	ND		ND		ND		ND		ND		ND	
SFA	16.19	0.48 ^d^	16.72	0.10 ^e^	13.01	0.33 ^c^	10.05	0.15 ^b^	9.66	0.06 ^a^	10.15	0.33 ^b^
MUFA	29.65	1.14 ^e^	18.49	0.52 ^d^	15.53	0.87 ^b,c^	14.83	0.34 ^b^	11.25	0.08 ^a^	16.18	0.65 ^c^
PUFA	14.34	0.64 ^b^	24.95	0.54 ^e^	31.15	0.98 ^f^	15.73	0.29 ^c^	20.79	0.06 ^d^	13.01	0.42 ^a^
*n*-3	3.64	0.24 ^d^	0.14	0.07 ^a^	3.51	0.15 ^d^	1.96	0.07 ^b,c^	3.66	0.05 ^d^	0.30	0.04 ^b^
*n*-6	10.70	0.86 ^a^	24.80	0.60 ^e^	27.64	0.84 ^f^	13.78	0.27 ^c^	17.14	0.04 ^d^	12.71	0.42 ^b^
TFA	0.39	0.02 ^e^	0.44	0.01 ^f^	0.25	0.03 ^d^	0.12	0.02 ^a^	0.19	0.01 ^b^	0.22	0.01 ^c^
Total fat content	61.50	5.15 ^b^	60.60	1.37 ^b^	59.71	1.44 ^b^	40.74	1.69 ^a^	41.82	0.8 ^a^	40.29	3.29 ^a^

Different letters in the same row indicate significant differences (*p* < 0.05) among the mean values for the different brands. ND: Not detected; SFA: sum of all saturated fatty acids; MUFA: sum of all monounsaturated fatty acids; TFA: sum of all *trans* fatty acids.

**Table 4:** Fatty acid profile of French fries (g/100 g fat) for the five fast food outlets analyzed (mean and standard deviation).

Fatty Acid	Brand 1		Brand 2		Brand 3		Brand 4		Brand 5	
	**Mean**	**SD**	**Mean**	**SD**	**Mean**	**SD**	**Mean**	**SD**	**Mean**	**SD**
Caprylic 8:0	0.14	0.01 ^a^	0.16	0.02 ^a^	0.14	0.02 ^a^	0.23	0.11 ^b^	0.12	0.00 ^a^
Capric 10:0	0.65	0.52 ^a^	0.10	0.02 ^a,b^	0.09	0.01 ^a^	0.14	0.07 ^b^	0.09	0.00 ^a,b^
Lauric 12:0	0.04	0.01 ^a,b^	0.09	0.01 ^c^	0.07	0.01 ^c^	0.05	0.02 ^b^	0.02	0.00 ^a^
Myristic 14:0	0.07	0.01 ^a^	0.31	0.04 ^c^	0.28	0.06 ^c^	0.19	0.05 ^b^	0.07	0.00 ^a^
Palmitic 16:0	5.08	0.35 ^a^	15.48	1.73 ^d^	13.44	2.37 ^c^	10.81	0.10 ^b^	5.27	0.22 ^a^
*t*-Palmitoleic 9*t*-16:1	ND		ND		ND		ND		ND	
Palmitoleic 9*c*-16:1	0.11	0.00 ^a^	0.14	0.01 ^b^	0.14	0.01 ^b^	0.13	0.02 ^b^	0.14	0.02 ^b^
Stearic 18:0	3.48	0.09 ^b^	4.01	0.01 ^c^	3.92	0.04 ^c^	4.31	0.06 ^d^	3.21	0.23 ^a^
Σ *trans* 18:1	0.21	0.05 ^a,b^	0.30	0.05 ^b^	0.25	0.02 ^a,b^	0.28	0.20 ^a,b^	0.19	0.02 ^a^
Oleic 9*c*-18:1	63.45	3.24 ^a^	63.59	0.74 ^a,b^	66.18	2.12 ^b^	30.08	3.04 ^c^	63.30	0.84 ^a^
11*c*-18:1	0.83	0.02 ^a^	0.81	0.02 ^a^	0.83	0.02 ^a^	0.78	0.06 ^a^	0.92	0.07 ^b^
*t*-Linoleic 9*t*, 12*t*-18:2	0.01	0.01 ^a^	0.01	0.00 ^a^	0.01	0.00 ^a^	0.04	0.01 ^b^	0.01	0.00 ^a^
*c-t* Linoleic 9*c*, 12*t*-18:2	0.14	0.01 ^b,c^	0.12	0.01 ^a,b^	0.11	0.00 ^a^	0.30	0.03 ^d^	0.16	0.02 ^c^
*t-c* Linoleic 9*t*, 12*c*-18:2	0.14	0.00 ^b^	0.13	0.01 ^a,b^	0.11	0.00 ^a^	0.27	0.04 ^c^	0.15	0.02 ^b^
Linoleic 9*c*,12*c*-18:2	24.79	2.52 ^b^	13.12	1.43 ^a^	12.70	0.51 ^a^	51.03	5.35 ^c^	24.66	0.83 ^b^
Arachidic 20:0	0.19	0.03 ^a^	0.27	0.03 ^b^	0.28	0.01 ^b^	0.15	0.11 ^a^	0.18	0.02 ^a^
γ-Linolenic 18:3*n*-6	ND		ND		ND		ND		ND	
Eicosenoic 20:1	0.18	0.01 ^c^	0.16	0.01 ^b^	0.18	0.01 ^c^	0.12	0.01 ^a^	0.18	0.01 ^c^
α-Linolenic 18:3*n*-3	0.19	0.01 ^a^	0.27	0.00 ^d^	0.24	0.03 ^b,c^	0.25	0.03 ^c,d^	0.22	0.00 ^b^
Eicosadienoic 20:2	ND	ND	ND	ND	ND					
Behenic 22:0	0.55	0.41 ^a^	0.64	0.06 ^a,b^	0.72	0.07 ^a,b^	0.61	0.11 ^a,b^	0.77	0.04 ^c^
Brassidic 13*t*-22:1	ND		ND		ND		ND		ND	
Erucic 22:1	ND		ND		ND		ND		ND	
Eicosatrienoic 20:3*n*-3	ND		ND		ND		ND		ND	
Arachidonic 20:4*n*-6	ND		ND		ND		ND		ND	
Eicosapentaenoic 20:5*n*-3	0.30	0.03 ^c,d^	0.25	0.02 ^b^	0.27	0.02 ^b,c^	0.21	0.05 ^a^	0.33	0.01 ^d^
Nervonic 24:1	ND		ND		ND		ND		ND	
Docosatrienoic 22:3	ND		ND		ND		ND		ND	
Docosapentaenoic 22:5*n*-6	ND		ND		ND		ND		ND	
Lignoceric 24:0	ND		ND		ND		ND		ND	
Docosapentaenoic 22:5*n*-3	ND		ND		ND		ND		ND	
Docosahexaenoic 22:6*n*-3	ND		ND		ND		ND		ND	
SFA	9.62	0.78 ^a^	21.08	1.74 ^d^	18.96	2.43 ^c^	16.50	2.04 ^b^	9.74	0.06 ^a^
MUFA	64.58	3.27 ^b^	64.70	0.73 ^b,c^	67.33	2.11 ^c^	31.12	3.10 ^a^	64.54	0.85 ^b^
PUFA	25.29	2.54 ^b^	13.65	1.43 ^a^	13.21	0.50 ^a^	51.48	5.32 ^c^	25.20	0.83 ^b^
*n*-3	0.49	0.04 ^a,b^	0.52	0.02 ^b,c^	0.51	0.02 ^b,c^	0.45	0.06 ^a^	0.55	0.02 ^c^
*n*-6	24.79	2.52 ^b^	13.12	1.42 ^a^	12.71	0.51 ^a^	51.03	5.35 ^c^	24.65	0.83 ^b^
*n*-6/*n*-3	50.63	4.93 ^b^	25.04	2.29 ^a^	25.07	1.81 ^a^	114.96	28.64 ^c^	45.00	2.43 ^b^
PUFA + MUFA/SFA	9.40	0.85 ^c^	3.75	0.39 ^a^	4.33	0.69 ^a^	5.10	0.83 ^b^	9.21	0.06 ^c^
TFA	0.51	0.06 ^a^	0.58	0.06 ^a^	0.49	0.02 ^a^	0.89	0.28 ^b^	0.51	0.05 ^a^

Different letters in the same row indicate significant differences (*p* < 0.05) among the mean values for the different brands; ND: Not detected; SFA: sum of all saturated fatty acids; MUFA: sum of all monounsaturated fatty acids; TFA: sum of all *trans* fatty acids.

**Table 5:** Fatty acid profile (g/100 g product) and total fat content (g/100 g product) of French fries for the five brands analyzed (mean and standard deviation).

Fatty Acid	Brand 1		Brand 2		Brand 3		Brand 4		Brand 5	
	**Mean**	**SD**	**Mean**	**SD**	**Mean**	**SD**	**Mean**	**SD**	**Mean**	**SD**
Caprylic 8:0	0.02	0.00 ^b^	0.01	0.00 ^a,b^	0.01	0.00 ^a^	0.03	0.01 ^c^	0.01	0.00 ^a^
Capric 10:0	0.01	0.01 ^a^	0.01	0.00 ^a^	0.01	0.00 ^a^	0.02	0.01 ^b^	0.01	0.00 ^a^
Lauric 12:0	0.00	0.00 ^a^	0.01	0.00 ^b^	0.01	0.00 ^b^	0.01	0.00 ^b^	0.00	0.00 ^a^
Myristic 14:0	0.01	0.00 ^a^	0.03	0.00 ^c^	0.02	0.00 ^b^	0.02	0.01 ^b^	0.01	0.00 ^a^
Palmitic 16:0	0.74	0.43 ^d^	1.45	0.23 ^b^	1.10	0.12 ^a^	1.32	0.20 ^b^	0.55	0.01 ^c^
*t*-Palmitoleic 9*t*-16:1	ND		ND		ND		ND		ND	
Palmitoleic 9*c*-16:1	0.02	0.00 ^c^	0.01	0.00 ^a,b^	0.01	0.00 ^a^	0.02	0.00 ^c^	0.01	0.00 ^b^
Stearic 18:0	0.51	0.00 ^c^	0.37	0.02 ^b^	0.33	0.03 ^a^	0.53	0.03 ^c^	0.34	0.04 ^a^
Σ *trans* 18:1	0.03	0.01 ^a,b^	0.03	0.00 ^a,b^	0.02	0.00 ^a,b^	0.03	0.02 ^b^	0.02	0.00 ^a^
Oleic 9*c*-18:1	9.25	0.58 ^c^	5.91	0.27 ^b,c^	5.52	0.78 ^b^	3.68	0.25 ^a^	6.59	0.32 ^c^
11*c*-18:1	0.12	0.00 ^d^	0.07	0.00 ^b^	0.07	0.01 ^a^	0.09	0.00 ^c^	0.09	0.00 ^c^
*t*-Linoleic 9*t*, 12*t*-18:2	ND		ND		ND		ND		ND	
*c-t* Linoleic 9*c*, 12*t*-18:2	0.02	0.00 ^b^	0.01	0.00 ^a^	0.01	0.01 ^a^	0.04	0.00 ^c^	0.02	0.00 ^b^
*t-c* Linoleic 9*t*, 12*c*-18:2	0.02	0.00 ^d^	0.01	0.00 ^b^	0.01	0.00 ^a^	0.03	0.00 ^e^	0.02	0.00 ^c^
Linoleic 9*c*,12*c*-18:2	3.61	0.32 ^c^	1.21	0.08 ^a^	1.06	0.16 ^a^	6.28	0.89 ^d^	2.57	0.17 ^b^
Arachidic 20:0	0.03	0.00 ^a,b^	0.02	0.00 ^a,b^	0.02	0.00 ^a,b^	0.02	0.01 ^a^	0.02	0.00 ^a^
γ-Linolenic 18:3*n*-6	ND		ND		ND		ND		ND	
Eicosenoic 20:1	0.03	0.00 ^c^	0.01	0.00 ^a^	0.01	0.00 ^a^	0.01	0.00 ^a^	0.02	0.00 ^b^
α-linolenic 18:3*n*-3	0.03	0.00 ^c^	0.02	0.00 ^b^	0.02	0.00 ^a^	0.03	0.00 ^d^	0.02	0.00 ^b^
Eicosadienoic 20:2	ND		ND		ND		ND		ND	
Behenic 22:0	0.08	0.06 ^a^	0.06	0.00 ^a^	0.06	0.01 ^a^	0.07	0.01 ^a^	0.08	0.01 ^a^
Brassidic 13*t*-22:1	ND		ND		ND		ND		ND	
Erucic 22:1	ND		ND		ND		ND		ND	
Eicosatrienoic 20:3*n*-3	ND		ND		ND		ND		ND	
Arachidonic 20:4*n*-6	ND		ND		ND		ND		ND	
Eicosapentaenoic 20:5*n*-3	0.04	0.00 ^c^	0.02	0.00 ^a^	0.02	0.00 ^a^	0.02	0.00 ^a^	0.03	0.00 ^b^
Nervonic 24:1	ND		ND		ND		ND		ND	
Docosatrienoic 22:3	ND		ND		ND		ND		ND	
Docosapentaenoic 22:5*n*-6	ND		ND		ND		ND		ND	
Lignoceric 24:0	ND		ND		ND		ND		ND	
Docosapentaenoic 22:5*n*-3	ND		ND		ND		ND		ND	
Docosahexaenoic 22:6*n*-3	ND		ND		ND		ND		ND	
SFA	1.40	0.11 ^b^	1.96	0.26 ^c,d^	1.56	0.11 ^b,c^	2.01	0.19 ^d^	1.01	0.06 ^a^
MUFA	9.42	0.59 ^d^	6.01	0.28 ^b^	5.61	0.78 ^b^	3.80	0.26 ^a^	6.72	0.31 ^c^
PUFA	3.68	0.32 ^c^	1.26	0.07 ^a^	1.10	0.17 ^a^	6.33	0.89 ^d^	2.63	0.17 ^b^
*n*-3	0.07	0.00 ^d^	0.05	0.00 ^b^	0.04	0.00 ^a^	0.06	0.01 ^c^	0.06	0.00 ^c^
*n*-6	3.61	0.32 ^c^	1.21	0.07 ^a^	1.06	0.16 ^a^	6.28	0.89 ^d^	2.57	0.17 ^b^
TFA	0.07	0.01 ^b^	0.05	0.00 ^a^	0.04	0.00 ^a^	0.11	0.03 ^c^	0.05	0.00 ^a^
Total fat content	14.58	0.28 ^e^	9.29	0.50 ^b^	8.32	1.04 ^a^	12.26	1.05 ^d^	10.42	0.70 ^c^

Different letters in the same row indicate significant differences (*p* < 0.05) among the mean values for the different brands; ND: Not detected; SFA: sum of all saturated fatty acids; MUFA: sum of all monounsaturated fatty acids; TFA: sum of all *trans* fatty acids.

## Supplementary Materials

The following are available online at www.mdpi.com/2072-6643/9/7/662/s1, Figure S1: Partial GC chromatogram showing a typical margarine FA profile, Figure S2: Partial GC chromatogram showing a typical French fries FA profile.

## Abbreviations

TFA	*trans* fatty acids
SFA	Saturated fatty acids
PUFA	Polyunsaturated fatty acids
MUFA	Monounsaturated fatty acids
