# Supplementary material for: Margarines and Fast-Food French Fries: Low Content of trans Fatty Acids
Source: Nutrients. 2017 Jun 28;9(7):662. doi: 10.3390/nu9070662 (PMC5537781; doi:10.3390/nu9070662)
Supplement: Supplementary file 1 [file nutrients-09-00662-s001.docx]

Figure S1. Partial GC chromatogram showing a typical margarine FA profile

1

2

3

5

6

8

9

11

15

13, 14

16

19

18

17

20

10

7

4

Figure S2. Partial GC chromatogram showing a typical French fries FA profile

3

4

6

7

8

9

11

10

15

16

19

18

20

12,13, 14

21

Codes for peaks: 1 (10:0); 2 (12:0); 3(14:0); 4 (16:0); 5 (9t-16:1); 6 (9c-16:1); 7 (internal standard); 8 (18:0); 9 (Σ t-18:1); 10 (9c-18:1); 11 (11c-18:1); 12 (9t, 12t-18:2); 13 (9c, 12t-18:2); 14 (9t, 12c-18:2); 15 (9c, 12c-18:2); 16 (20:0); 17 (18:3n-6); 18 (11c-20:1); 19 (18:3n-3); 20 (22:0); 21 (20:5n-3).
